# The Institutional Library

**Published:** 1917-05-05

**Authors:** 


### THE INSTITUTIONAL LIBRARY.

**The Biology of Tumours.** By C. MANSELL MOULLIN, M.A., M.D. Oxon, F.R.C.S. (London: H. K. Lewis and Co., Ltd. 1916. Demy 8vo. Pp. 55. Price 2s. 6d. net.)

THIS is a short essay on the ætiology of tumours written by a surgeon who is also a biologist. It is an elaboration of the Bradshaw Lecture given at the Royal College of Surgeons of England in 1912. The theses to be defended are that tumours fall into two classes according to their mode of origin. "One of these is due to the sudden awakening of the innate reproductive power of the tissues, in virtue of which they give birth to buds that grow into tumours. The other is due to details of structure not being carried out as completely as they ought to be." Mr. Moullin has given much thought to both modes of origin, but he is evidently fascinated by the first, and explains some tumour formations as being due to the persistence of the power in cells of forming buds from which the tumour originates. The result of the persistence of bud formation or asexual gemmation varies with the condition of the cell. If the cells, influenced by some irritant—whether mechanical, chemical, or physiological—have all but reached adult age before the interference is felt, the buds that grow from them are all but adult too. The tumour is then composed of tissues that resemble those of the normal skin. But if, owing to the condition, there is a great increase in the proportion of young rapidly-growing cells and the development of these is checked in their youth, the buds that spring from them resemble them, and then the tumour increases rapidly and spreads wherever it can. The development of the tissues is said to depend upon special chemical reactions which have been evolved in the course of ages and are still being evolved. The whole question is very speculative, but it could be put to the test of experiment, as might easily be done.

**Notes on Military Orthopædics.** By Colonel ROBERT JONES, C.B. (London: Cassell and Co. Pp. 132. Price 2s. 6d. net.)

THIS slim volume by the greatest British expert of the day on the subject dealt with is well worthy of his high reputation and of British surgery. It should be in the haversack of every medical officer who has to treat cases of gunshot wounds, either at the Front, the Base, or at home. The present writer has worked in medical units ever since the outbreak of the war, and thought he had gained a reasonably full knowledge of the treatment of wounds; but he is absolutely horrified to discover how many facts he has hitherto been ignorant of, and what a lot of Colonel Jones's teaching is novel to him. There need be no hesitation on the part of any doctor in admitting that this short and simple manual puts the subject of military orthopædics in a form which illumines afresh every branch of that intricate subject. Even if the author had done nothing else but write this book he would have deserved well of the Army and the State. In common with other authors, Colonel Jones, it may be observed, uses the term "dorsi flexion" (of the wrist) for what anatomists call "extension" of that joint. The point is of small importance, provided the meaning of the term is clear, as in this case it is. Another point of nomenclature is that when Colonel Jones speaks of an elbow joint flexed "at 70°" he does not mean that that angle subsists between the lines of the forearm and upper arm, but that the forearm has been bent 70° from its normal position of full extension. In other words, the angle formed at the elbow is 110°—an obtuse angle, not an acute one. The diagrams make it clear what is meant; but without them the wording is susceptible of misconstruction.

**Songs of Protest.** By FREDERIC L. MITCHELL. (Erskine Macdonald. 1s. net.)

IN his interesting introduction to these verses, Mr. R. O. Prowse, the novelist, describes their writer as a "poet of the under-world," and claims originality for him on the ground that his realistic studies of slum life do not allow his readers "to take refuge in the redeeming trait." His blackness is unrelieved. That is exactly why the verses are not convincing. The slum-dweller finds plenty of relief: in the blows, drink, petty chicaneries, crime, and lusts which to middle-class persons seem unrelievedly dreadful. The attitude of the degraded poor is one of pure cynicism, which rather glories in than revolts from the brutality of their lives. If they had not the escape of cynicism they could not endure their lives at all; and therefore a poet who would study them from within must portray this view if his characters are to live for us. As it is, the point of view of the square, turning away in horror from the slum, is really what Mr. Mitchell portrays, and that is why we call his slum studies superficial. The correctness of this view can be shown by comparing his verses with those of Mr. W. H. Davies, the super-tramp, who knows his subject at first-hand. There is certainly no veiling of "realism" in *his* slum studies, but there are no middle-class regrets either. The humour, the cynicism, the brutality stand out as living forces. Mr. Mitchell gives the show away in his very title, "Songs of Protest"—that is, the middle-class point of view. He should have called them "Songs of Exuberance," had not that admirable title been already appropriated by a volume which appeared last year. Let him also realise that the mechanical lilt of most of his verses is wearisome, and ask himself why it is that almost every line taken by itself stands revealed as merely bold and prosaic, without a note of music anywhere, except in the "Viking of Ludgate Hill." The technical reason for this is the absence of vowel sounds, which explains, for instance, why "A Mr. Wilkinson, a clergyman," is the stock example of the worst blank-verse line in the language. The real "redeeming trait" in sordid surroundings is the energy, expressing itself in humour, brutality, or boozing, which make the men who live in them so much a part of their environment that they are unconscious of everything except the needs and feelings of the moment. This is what Mr. Mitchell's studies at present lack; and it is Mr. Prowse's confusion of this with the sentiment invoked by inferior writers to "redeem" their coarse scenes which makes him remark that "Truth is . . . perhaps impossible to tell in most forms of imaginative expression." Truth cannot be told in any other form, and that is why there is more truth in fairy tales than in the Post Office Directory, or in last night's newspaper. For a "realistic" writer to print w—— to rhyme with floor is to convict himself of the worst kind of prudery; that is to say, of being ashamed of his own convictions. Perhaps this was the publisher's doing, however. That is the sort of mistake which a publisher makes.

**Studies in Malaria.** By Hugh Stott, Captain I.M.S. In Five Parts. (Calcutta and Simla : Thacker, Spink and Co. 1916.)

This treatise on malaria contains a number of personal observations by the author, chiefly made between July 1911 and February 1913, when he was quartered in Mandalay on military duty. In addition, further clinical experiences are incorporated. One of the most interesting chapters of the book is that dealing with an inquiry into the results of twelve months' supervised issue of prophylactic quinine to one half of a regiment, the remaining half serving as a control. The results were very disappointing; the use of the drug did not in any way prevent the disease, and exerted no practical influence on the severity of a subsequent malarial attack. This experience is in keeping with similar results obtained by Linell and others, and it shows that, where one is being constantly bitten by infected mosquitoes, the drug will not save one.

Captain Stott has also some very interesting remarks to make on the use of quinine in treatment, and deals with the vexed question of injections *versus* the oral route. He shows that in some instances the drug will not act satisfactorily when given by the mouth alone, and in these cases it has to be given by the needle intramuscularly, or even into the veins direct; the latter method is the safer in pernicious types where speed is of importance. Conversely, in some instances injections do not do well if used alone, and have to be supplemented by the mouth. The results of salvarsan were disappointing, but the use of arsenic by the mouth in the cure of chronic cachectic patients is properly mentioned, and due stress is laid upon this useful auxiliary to quinine.

The book is very well illustrated with charts, maps, and photographs. It certainly contains much useful first-hand information, and the author is to be congratulated upon its production.

**Broca's Ligations and Amputations.** Translated by Ernest Ward, M.D., F.R.C.S. (Bristol : John Wright and Sons, Ltd. Price 8s. 6d. net.)

This is a translation of a work entitled "Précis de Médecine Operatoire," by Professor Broca, of the Faculté de Médecine de Paris. The title given by the translator is, to our mind, wisely selected, as it connotes the sphere of the work in a more precise manner than does the original one. The author states that the book is written only for students who take the knife in hand for the first time; but we think that it should appeal to more advanced students, especially those who are preparing for the higher surgical examinations, and the young hospital surgeon may find in it much that will be helpful to him in his daily work, for Professor Broca emphasises the importance of the guiding principles in performing amputations, which have often been lost sight of in the publications of more recent years, and also gives much information which is the fruit of his experience in the present war.

The volume is divided into two sections. The ligation of arteries is first dealt with, great attention being paid to surface landmarks, and copious and clear anatomical drawings are added to the text, which facilitates the description of the operations.

The second section treats of amputations and disarticulations. Preliminary chapters on the use of instruments, the division of the skin, the shaping of stumps, and the choice of operation and of the artificial limb are all clear and full of practical points. Upon these follow the description of the various amputations, and here, too, the standard of the preceding part of the volume is maintained. Particularly noticeable and worthy of praise are the illustrations. The anatomical drawings are from Farabeuf's book on amputations, and from the wall diagrams which he used in his classes; and the illustrations of operative technique are specially executed by M. Reignier from stereoscopic photographs which the author himself produced. By means of these latter the student will have no difficulty in following the description in the text, and with sufficient practice on the cadaver should be able to acquire technical skill.

The translator is to be congratulated on having accomplished his task in a most satisfactory way, and has certainly produced a book which should be of value to English students.

**Pocket Anatomical Atlas of the Human Body.** Edited by a London M.D. (London : The Scientific Press. Price 1s. 3d. Nine coloured plates.)

This Atlas of coloured plates must surely be one of the cheapest really reliable compendiums of anatomy ever published. It is true that the octavo size of it reduces some of the smaller details in the plates to minute size; but the drawings are so accurate and clearly differentiated that there is no difficulty in identifying quite easily every single structure to which the numerous indicator-labels refer. This publication should be of the very greatest value to nurses, masseuses, V.A.D.s, or anyone training for those employments. The plates are splendidly produced, and the greatest trouble has evidently been taken to ensure the most rigid accuracy. The Berne anatomical nomenclature is used throughout.

**Common Diseases of the Male Urethra.** By Frank Kidd, F.R.C.S. (London : Longmans, Green and Co. Pp. 132. Price 5s. net.)

This is in many ways a somewhat unconventional book; but it is unquestionably fully informed by clinical wisdom and ripe experience. Mr. Kidd is not afraid to state his convictions forcibly, even when they run counter to some of the fads of the moment. We cannot imagine anything but good accruing to the medical student or practitioner from a study of these pages and an adoption of the general lines of treatment laid down in them. Here is specialism seen at its best, combined with a sound knowledge of general surgery and of basal surgical principles. Mr. Kidd knows what he means and is not afraid to say it; his remarks about vaccines are most refreshing.

**A Handbook of Midwifery for Nurses.** By Comyns Berkeley, M.D., F.R.C.S. (London : Cassell and Co. Fourth Edition. Pp. 528. Price 6s. net.)

Dr. Comyns Berkeley has made a good many alterations in and additions to this new edition of his well-known text-book for midwives, maternity nurses, and obstetric dressers. Finality and perfection are things that medical writings seldom, if ever, attain; but this particular book certainly gets so near them that one wonders where the author will find scope for improvements in his next edition. Clearness of thought, logical arrangement, and judicious selection of what to insert and what to omit in a small book of this aim are the leading characteristics of Dr. Berkeley's work. Incidentally, it is a rare thing to find a medical man who is equally happy in his elementary and in his advanced expositions of his particular branch of science.

